# Power Reduction in Punch-Through Current-Based Electro-Thermal Annealing in Gate-All-Around FETs

**DOI:** 10.3390/mi13010124

**Published:** 2022-01-13

**Authors:** Min-Kyeong Kim, Yang-Kyu Choi, Jun-Young Park

**Affiliations:** 1 School of Electronics Engineering, Chungbuk National University, Chungdae-ro 1, Chungbuk, Cheongju 28644, Korea; kming@chungbuk.ac.kr; 2 School of Electrical Engineering, Korea Advanced Institute of Science and Technology (KAIST), Daejeon 34141, Korea

**Keywords:** annealing, dielectric, gate-all-around (GAA), hot-carrier injection (HCI), power consumption, punch-through, reliability, logic transistors

## Abstract

Device guidelines for reducing power with punch-through current annealing in gate-all-around (GAA) FETs were investigated based on three-dimensional (3D) simulations. We studied and compared how different geometric dimensions and materials of GAA FETs impact heat management when down-scaling. In order to maximize power efficiency during electro-thermal annealing (ETA), applying gate module engineering was more suitable than engineering the isolation or source drain modules.

## 1. Introduction

MOSFETs have been aggressively scaled down to improve packing density and chip performance [1]. However, as semiconductor devices shrunk, several issues have arisen, such as short-channel effects (SCEs). SCEs give rise to an increase in the off-state current (*I*_OFF_) and subthreshold swing (*SS*) and result in an increase in static power consumption (*P*_OFF_ = *V*_DD_ × *I*_OFF_) in the OFF-state. SCEs have been effectively suppressed by improving gate controllability not only with three-dimensional (3D) device structures such as FinFETs and gate-all-around (GAA) FETs, but also high-k gate dielectric and metal gate (HKMG) technology. In contrast to SCEs, improving device reliability during device minimization has become increasingly difficult. For example, recently, gate dielectric damage from hot-carrier injection (HCI), which is associated with the lateral drain electric field, has resurfaced as a matter of concern in semiconductor devices [2,3]. Typically, HCI increases both the threshold voltage (*V*_T_) and *SS*, and hence results in unwanted *V*_T_ mismatching while also increasing *I*_OFF_ in circuitries. In addition, the HCI decreases both the ON-state current (*I*_ON_) and lifetime, which affect chip speed and long-term usability, respectively [4,5].

To overcome the degradation of the gate dielectric, lightly doped drains (LDD) or forming gas annealing (FGA) have been more commonly used in mass production for decades [6,7]. However, it is difficult to realize long-term reliability longer than 10 years. Hence, electro-thermal annealing (ETA), which utilizes local heat generated by the device itself, has been introduced as a novel approach to cure the damaged gate dielectric [8].

It is possible that gate dielectric damage resulting from various stresses such as ionizing radiation, bias temperature instability, and HCI can be healed with the aid of ETA [8]. However, even though ETA can improve a device’s reliability and lifetime, additional power consumption is inevitable, since ETA is performed by generating high-temperature Joule heating. To reduce power consumption an alternative is needed that would improve the power efficiency of ETA while enabling high-temperature generation.

In this work, the effects of geometric size and the material of the GAA FET were investigated to improve power efficiency during ETA. COMSOL simulation software was used to better understand the thermal dissipation and isolation characteristics during ETA. Temperature sensitivities were extracted and compared with respect to the gate module, including the gate electrode and gate spacer, source/drain module, and isolation layer.

## 2. Materials and Methods

**Figure 1:**
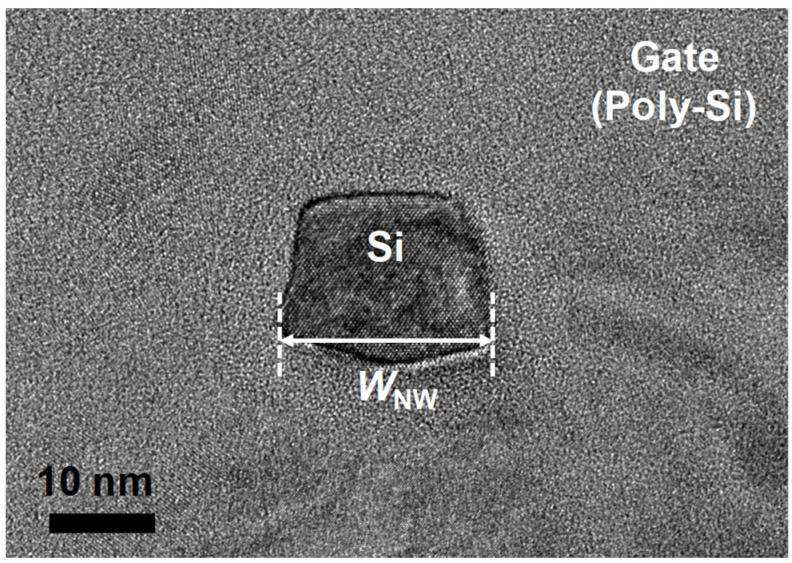
Transmission electron microscopy (TEM) image of the fabricated GAA FET.

Gate-all-around (GAA) FETs, fabricated on bulk wafer [9], as shown in Figure 1, were simulated as test specimens. The channel thickness (*T*_Si_), channel width (*W*_NW_), gate length (*L*_G_), and gate height (*H*_G_) were 20 nm, 20 nm, 60 nm, and 250 nm, respectively. The thickness of the gate hard mask (*T*_HM_) and gate spacer (*T*_SPC_), which are composed of SiO_2_, were 50 nm and 30 nm, respectively.

**Figure 2:**
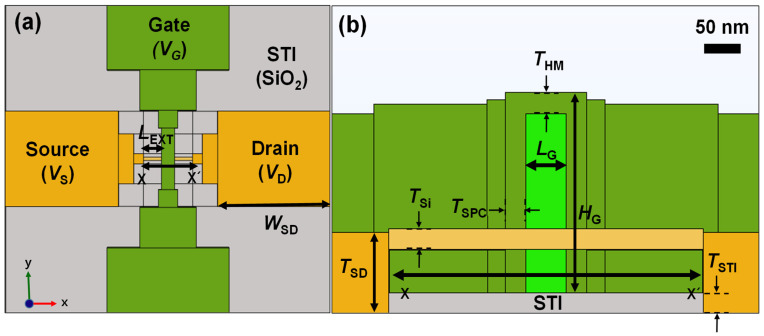
Schematic of the device used for simulations. (**a**) Top-view image of the GAA FET. (**b**) Cross-sectional image of the GAA FET cut along the x–x′ direction.

Figure 2 shows a schematic of a GAA FET built on a bulk substrate for simulations. The Joule heating model in the heat transfer module of COMSOL was applied for 3D thermal profiling. During the simulation, the environment state and heat transfer coefficient (*h*) were assumed to be air and 10 W/m^2^K, respectively. After that, punch-through current [10] was used for ETA instead of forward junction current [11] or gate-to-gate [12] current. Detailed device information used for the simulations are summarized in Table 1.

## 3. Results and Discussion

**Figure 3:**
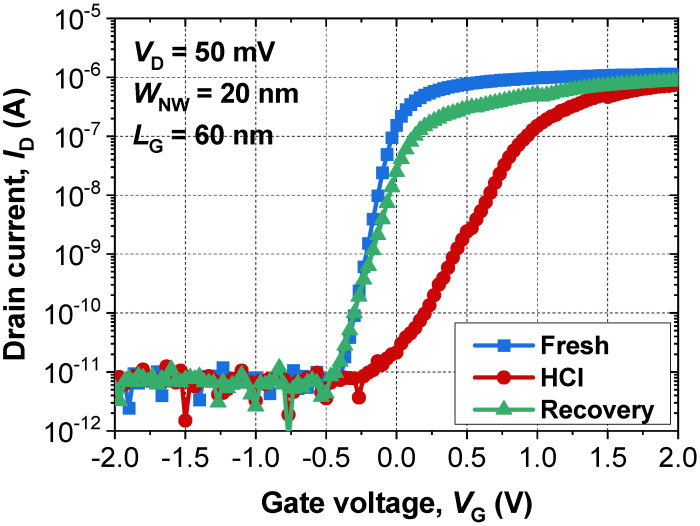
Measured *I*_D_-*V*_G_ characteristic of the fabricated n-channel GAA FET.

Figure 3 shows the measured electrical *I*_D_-*V*_G_ characteristics of the GAA FET. The DC characteristic was measured using a B1500A parameter analyzer at room temperature. After measurement of the initial state (e.g., initial state without stress), HCI stress at *V*_G_ = 2 V and *V*_D_ = 4 V was deliberately administered for 2 s. After the stress, degradation in the transconductance *SS* and *V*_T_ were observed at 227 mV/dec and 0.65 V, respectively. After that, bias conditions with *V*_G_ = 0.5 V and *V*_D_ = 6 V were applied for 100 μs to trigger a punch-through current-based ETA (Table 2). In fact, the current at the pinch-off is independent of *V*_G_, and the *V*_G_ of 0.5 V was just referenced from our previous work [10]. After ETA, the aged-device characteristics with respect to *SS* and *V*_T_ recovered by 124 mV/dec and −0.05 V, respectively, compared to the initial state (Table 3). These facts show that both electrons were trapped in the gate dielectric, and physical damage at the SiO_2_/Si interface was effectively cured by the punch-through current-based ETA.

**Figure 4:**
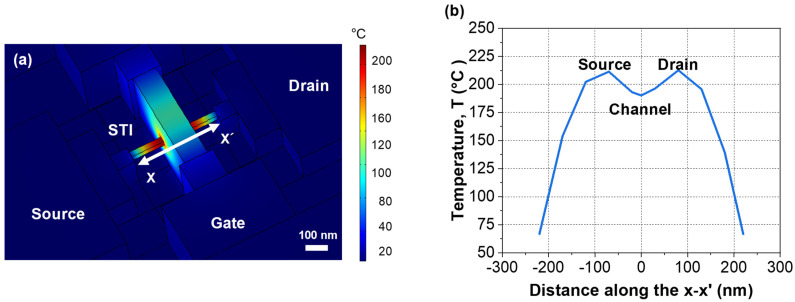
(**a**) Simulated heat distribution division profile during punch-through-based ETA under bias conditions with *V*_G_ = 0.5 V, *V*_S_. = 0 V, and *V*_D_ = 6 V. (**b**) Extracted temperature of the device along the x–x′ direction during ETA.

Figure 4 shows a simulated heat distribution profile during ETA driven by the punch-through current in Figure 2. It shows that most of the heat during ETA was concentrated at the source/drain (S/D) extension where gate heat sink could not affect it. The extracted temperature at the S/D was symmetric [13]. However, considering the self-heating effect of semiconductor devices, the drain temperature was higher than that of the source region [14]. 

**Figure 5:**
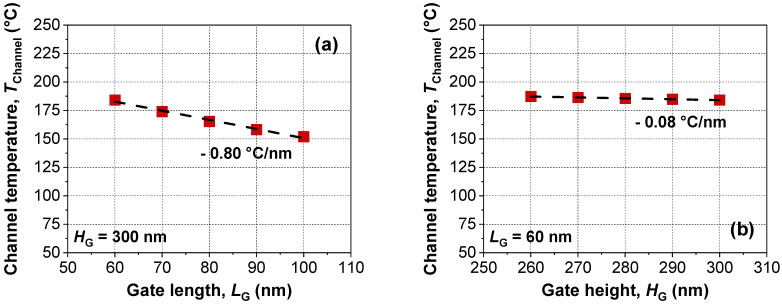
Extracted *T*_Channel_ of devices with various (**a**) gate lengths and (**b**) gate heights under an identical power consumption of 0.45 mW. Dashed lines indicate linear fits of the experimental data.

Figure 5 shows the extracted channel temperature (*T*_Channel_) with respect to gate electrode scaling. All temperatures were extracted at the center of the silicon nanowire channel, i.e., *L*_G_/2.

As the physical gate length and the height of the device were reduced, the temperature during ETA increased. Typically, the gate electrode acts as the heat sink during ETA. As the volume of the gate decreased, the temperature during ETA increased due to the reduced heat sink. The consistent high temperature generated during ETA under identical applied power consumption represented better power efficiency for gate dielectric curing. In this context, considering the extracted sensitivity of temperatures with respect to the gate length and the height, it would be more efficient to apply gate length scaling rather than the gate height. In addition, the gate module includes not only the gate electrode itself but also dielectric materials such as gate dielectric, gate spacer, and gate hard mask.

**Figure 6:**
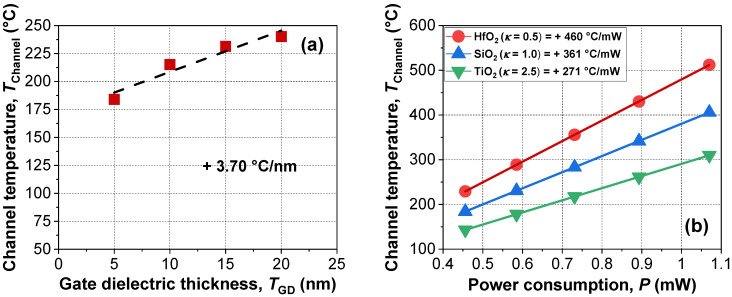
Extracted *T*_Channel_ of devices with various (**a**) thicknesses and (**b**) materials of gate dielectric. Dashed lines indicate linear fits of the experimental data.

Figure 6a shows the extracted *T*_Channel_ with various thicknesses of gate dielectric composed of SiO_2_. As the gate dielectric thickness (*T*_GD_) increased, channel temperature increased under identical power consumption due to decreased heat dissipation through the gate electrode. However, considering the gate dielectric thickness was scaled down for better suppression of SCEs, this approach seems impractical for reducing power consumption. Alternatively, the material engineering shown in Figure 6b would be more efficient. As the thermal conductivity (*κ*) of the gate dielectric decreased, temperature sensitivity with applied power increased, due to increased thermal isolation, i.e., reduced heat dissipation with low *κ*.

**Figure 7:**
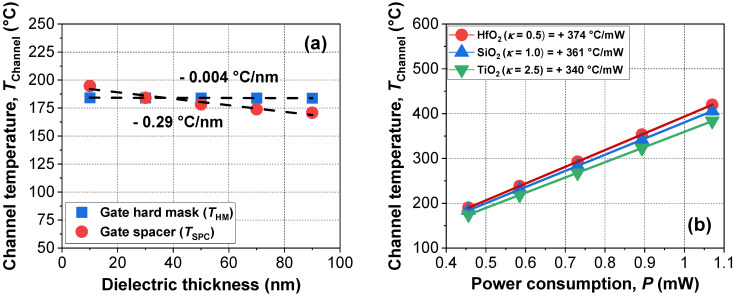
Extracted *T*_Channel_ of devices with various (**a**) dielectric thicknesses and (**b**) materials of gate hard mask and gate spacer. Dashed lines indicate fitting of the symbols.

Figure 7a shows the extracted *T*_Channel_ with various dielectric thicknesses of gate hard mask (*T*_HM_) and gate spacer (*T*_SPC_). The *T*_HM_ had a negligible effect on *T*_Channel_ compared with the gate dielectric engineering in Figure 6. As the gate spacer increased, the temperature during ETA decreased due to the increased surface area of the gate spacer. Since convective cooling is performed through the air, a gate spacer with a small width and surface area would be more preferred to lower power consumption. Figure 7b shows the extracted channel temperature with various levels of thermal conductivity for the gate hard mask and the spacer. As the thermal conductivity of the dielectrics decreased, temperature sensitivity increased due to increased thermal isolation.

**Figure 8:**
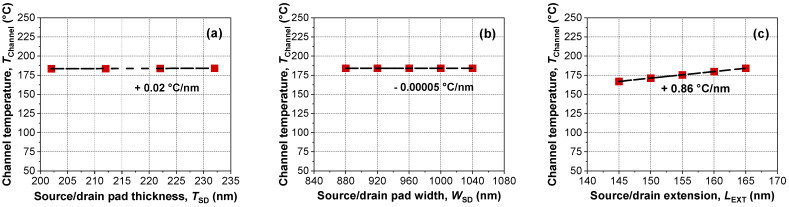
Extracted *T*_Channel_ of devices with various (**a**) S/D pad thicknesses, (**b**) S/D pad widths, and (**b**,**c**) S/D extension lengths. Dashed lines indicate linear fits of the experimental data.

In contrast to the results in Figure 5 to Figure 7, which focused on the gate module, Figure 8 shows the device temperature with respect to modifications of the S/D module. However, even though the S/D extension showed the largest temperature sensitivity (Figure 8c), the sensitivity stemming from S/D was negligible. Moreover, considering the S/D extension (*L*_EXT_) had been scaled down for better packing density, this approach seems impractical. In this context, reducing power consumption by engineering of the S/D module is not recommended.

**Figure 9:**
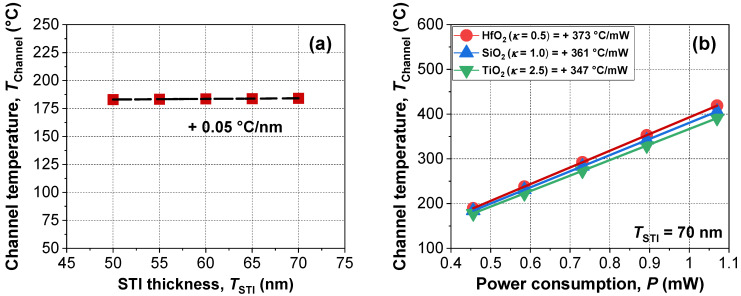
Extracted *T*_Channel_ of devices with various (**a**) thickness and (**b**) materials of shallow trench isolation (STI). Dashed lines indicate linear fits of the experimental data.

Figure 9a shows the extracted *T*_Channel_ in the case of the isolation engineering by use of shallow trench isolation (STI) technology. As thickness *T*_STI_ increased, channel temperature could be increased due to the increased thermal isolation. However, the change was negligible because the channel was suspended from the STI. Figure 9b shows power efficiency with various buried dielectric materials. When a low thermal conductive material, e.g., HfO_2_, is employed instead of SiO_2_, the channel temperature could be increased under identical power consumption. Based on these results, our recommendation to maximize power efficiency is to apply low thermally conductive materials as an STI.

Table 4 provides a summary of the temperature sensitivities for the different geometries and materials of the GAA FET. It can be concluded that the most significant design parameter for determining power efficiency is gate module engineering. As a result, the approach using gate module engineering would be more preferred to reducing power consumption for punch-through current-based ETA.

## 4. Conclusions

Device guidelines for reducing the power of punch-through current annealing were investigated using 3D COMSOL simulations. Power management efficiency can be improved with dimensional and material engineering. The impacts of device scaling with respect to gate module, source/drain (S/D) module, and isolation, were compared in detail. The gate module engineering was found to be the most significant way to reduce power consumption. However, in contrast to the gate module, impacts of the S/D and shallow trench isolation were negligible.

## Figures and Tables

**Table 1:** Dimensional and material parameters for COMSOL simulations.

Geometry	Dimension	Material	Thermal Conductivity [W/m∙K]
Gate length, *L*_G_ [nm]	60	Poly-Si	31.2
Gate height, *H*_G_ [nm]	300		
Gate hard mask thickness, *T*_HM_ [nm]	30	SiO_2_	1
Gate spacer thickness, *T*_SPC_ [nm]	30		
Gate dielectric thickness, *T*_GD_ [nm]	5		
STI thickness, *T*_STI_ [nm]	70		
Source/drain pad thickness, *T*_SD_ [nm]	232	Si	149
Source/drain pad width, *W*_SD_ [nm]	1040		
Channel thickness, *T*_Si_ [nm]	20		
Channel width, *W*_NW_ [nm]	20		
Source/drain extension length, *L*_EXT_ [nm]	165		

**Table 2:** Bias conditions for punch-through current based ETA.

	Bias Condition
Gate voltage (*V*_G_)	0.5 V
Source voltage (*V*_S_)	0 V
Drain voltage (*V*_D_)	6 V
Punch-through current (*I*_Punch_)	75 μA
Power consumption, (*P* = *V*_D_ × *I*_Punch_)	0.45 mW
Annealing time (*t*)	100 μs

**Table 3:** Extracted device parameters before HCI, after HCI and ETA.

	Initial State (Before HCI)	After HCI	After Punch-Through ETA
*SS* (mV/dec)	82 mV/dec	227 mV/dec	124 mV/dec
*V*_T_ (V)	−0.13 V	0.65 V	−0.05 V

**Table 4:** Summary of temperature sensitivity according to dimensional and material engineering of the gate, S/D, and isolation module for the punch-through current-based local thermal annealing.

	Gate Module	S/D Module	Isolation
Minimum (°C/nm)	−0.80	0.00	+0.05
Maximum (°C/nm)	+3.70	+0.86	
